# SARS-CoV-2 exposure in hunting and stray dogs of southern Italy

**DOI:** 10.1007/s11259-024-10496-9

**Published:** 2024-08-21

**Authors:** Gianmarco Ferrara, Ugo Pagnini, Serena Montagnaro

**Affiliations:** Department of Veterinary Medicine and Animal Productions, University of Naples Federico II, Via Federico Delpino n.1, Naples, 80137 Italy

**Keywords:** Hunting dogs, Stray dogs, SARS-CoV-2, Coronavirus

## Abstract

Evidence of exposure to the pandemic SARS-CoV-2 has been described in numerous animal species, including pets, which are predisposed to coming into contact with this virus due to their close relationship with owners. It has been accepted that dogs are poorly susceptible to this virus and that seroconversion, rather than shedding, occurs following infection, which can occur directly through contact with infected owners or indirectly through environmental contamination. In this study, the seroprevalence of SARS-CoV-2 was evaluated in apparently health hunting and stray dogs of Campania region, southern Italy (sampled in September 2023). A total of 5/112 (4.5%) animals tested seropositive using two different commercial ELISAs. Stray animals had greater exposure than hunting dogs. The feces and blood of each animal were tested with a real-time PCR targeting the nucleocapsid and ORF1ab coding sequences. No animal tested positive in molecular investigations, indicating a past exposure without active infection at the time of sampling.

## Introduction

Severe acute respiratory syndrome coronavirus 2 (SARS-CoV-2) is a single-stranded RNA virus responsible for the COVID-19 pandemic in humans (Jamison et al. 2022). The infection has caused billions of cases worldwide, millions of deaths, as well as huge economic losses. The scientific community was immediately concerned about possible spillovers towards animals (Zhao et al. 2024). SARS-CoV-2 has been described in cats, dogs, non-human primates, minks, and nowadays in more than 30 animal species (including livestock, wildlife, and zoo animals) (Bartlett et al. 2021; Fiorito et al. 2022; Frazzini et al. 2022; Teixeira et al. 2023). Intraspecies transmission has also been demonstrated in some animals (Frazzini et al. 2022).

Dogs and cats are highly predisposed to SARS-CoV-2 exposure because of their direct interaction with infected owners (Murphy and Ly 2021; Decaro et al. 2021a; Fischer et al. 2023). Experimental airborne infection in dogs could result in viral excretion through the feces (although internal organs test negative even 4 days after infection) and the development of a measurable antibody response 14 days later (Patterson et al. 2020; Pourbagher-Shahri et al. 2023; Tomeo-Martín et al. 2024). Evidence in the literature suggests that antibodies are detectable in animals up to approximately 10 months after exposure (Decaro et al. 2021b). In some cases, the virus replicates in the mucosal system of the oropharynx and nasopharynx (Frazzini et al. 2022). The majority of affected dogs exhibited no symptoms and had low viral loads, with some exceptions. In 2020, a case of diffuse and severe pulmonary interstitial pattern was described in a 3-year-old female bull terrier in Italy, which was serologically positive for SARS-CoV-2 (Colitti et al. 2022). In 2021, a case of human–dog SARS-CoV-2 transmission was described in a Scottish terrier in Spain, characterized by hemorrhagic enteritis that was negative for the main canine enteric pathogens. Subsequent sequencing of the spike region revealed the strain involved (Padilla-Blanco et al. 2022). It is now possible to assess the exposure of pets and other animals to SARS-CoV-2 using multispecies ELISAs that have been specifically designed (or adapted) for this purpose (Cavalera et al. 2021; Ratti et al. 2022).

Although SARS-CoV-2 prevalence in dogs has been extensively studied over different periods, there have been few studies that evaluate the dynamics of transmission across dogs in various living conditions (for example, hunting or stray dogs), as well as the associated risk factors for each. The purpose of this study was to assess the exposure of hunting dogs and stray dogs to SARS-CoV-2 in the Campania canine population of southern Italy, as well as the virus's potential spread in the environment via feces.

## Materials and methods

### Study area and sampling

The present study was conducted in the Campania region, southern Italy (40°49′34″N 14°15′23″E). Campania is the most populous and densely populated region in the South (5,597,358 inhabitants). Consequently, a large number of dogs live in this area, many of them stray or hunting dogs (mainly used for hunting wild boar, which is the main game species in the region) (Ferrara et al. 2022). Sampling began in September 2023, when in Italy the main circulating SARS-Cov-2 variant was omicron EG.5, and was completed in December 2023. For each animal, a blood sample was collected in a tube without anticoagulant (for serum) and a stool sample directly from the rectal ampulla (Ferrara et al. 2024b). After the collection, all samples were immediately transported to the Department of Veterinary Medicine and Animal Production in Naples, maintaining the cold chain. Each sample was accompanied by a document containing the animal's data (age, sex, breed, attitude, size, and location) (Ferrara et al. 2024a). The stool samples were stored at -80 °C. The blood samples were centrifuged, and the resulting serum was collected and stored at -20 °C. The study protocol was approved by the Institutional Ethics Committee of the Department of Veterinary Medicine and Animal production (Centro Servizi Veterinari), University of Naples, Federico II (PG/2022/0093420, 21st July 2022).

### Serological analysis and statistical strategy

All serum samples were tested for the presence of antibodies directed against the nucleocapsid (N) antigen and towards the spike (S) antigen using two ELISAs, respectively ID Screen® SARS-CoV-2 Double Antigen Multi-species ELISA (IDVet) and WANTAI SARS-CoV-2 Ab ELISA (WANTAI). Both tests were double-antigen ELISAs exploiting an antigen conjugate (SARS-CoV-2 N antigen-HRP conjugate, and receptor-binding domain of SARS-CoV-2 spike protein-conjugated) and are widely used for serosurveys in various animal species. The tests were carried out following the manufacturer's instructions. The reading of optical density (OD) values was performed with a spectrophotometer. The values obtained were compared with the cut-offs suggested by the manufacturers to evaluate the positivity of each sample. Chi-square statistics were used to analyze the association between the dependent (ELISA outcome) and independent factors (as obtained from the questionnaire).

### Molecular analysis

**Fig. 1:**
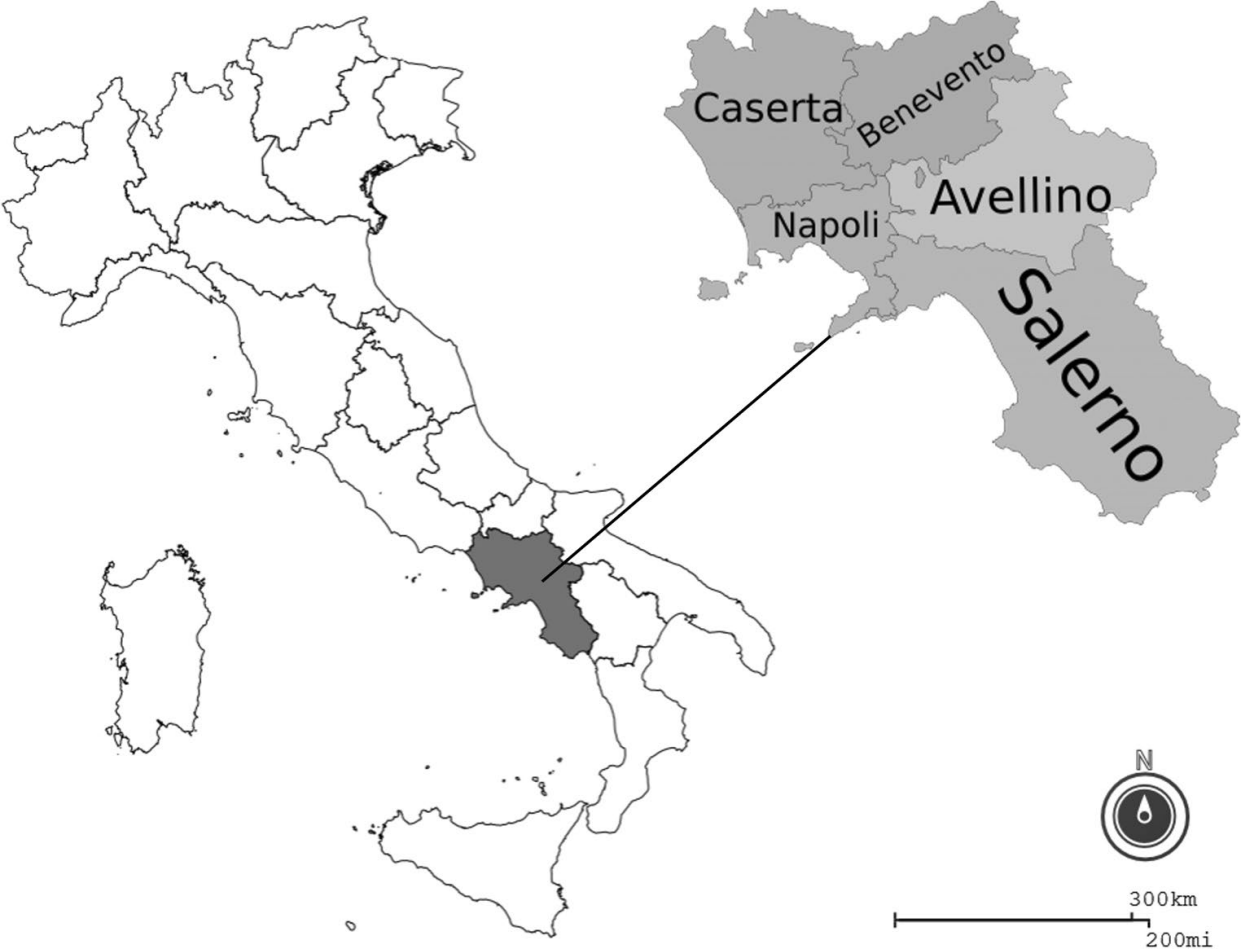
Map of the study area (Campania region, southern Italy) created using the geographic information system Epinfo

RNA from each fecal sample was extracted using the commercial kit QIAamp® Viral RNA Mini Kit (Qiagen). The extracted RNA was quantified by nanodrop and used as a template for a single-step-RT-real-time PCR (Norgen’s COVID-19 TaqMan RT-PCR Kit N/ORF1ab genes, Norgen) for simultaneous amplification of the N gene and the Open Reading Frame 1ab (ORF1ab). The amplification reaction included 2X One-Step RT-PCR Master Mix, appropriate primers and template RNA. The kit provided both the positive control for ORF1ab and N (included in the microplates). Thermal conditions included a reverse transcription step at 50 °C for 30 min, a denaturation step at 95 °C for 3 min followed by 45 cycles of amplification (95 °C for 3 s and 55 °C for 30 s). Results were read with a CFX96™ Real-Time PCR Detection System (Bio-rad) (Fig. 1).

## Results

A total of 112 dogs were sampled (53 hunting and 59 stray). The results highlighted the presence of antibodies against SARS-CoV-2 in 5 out of 112 dogs tested, corresponding to an overall seroprevalence of 4.5% (Table 1). The positive results were found to have the same outcome in the two tests used. Statistical investigation explored the possible correlation between different risk variables and greater seroprevalences. Although not statistically significant, seroprevalence was higher in certain locations (Benevento and Caserta) than in others (Avellino and Naples), where no animals tested positive. The same was true for breed and age. Crossbred animals showed more exposure (8.7%) than purebred animals (1.5%). Exposure to SARS-CoV-2 in dogs in the Campania region showed an age-related tendency (no young animals were seropositive, but 3.6% and 11.1% of adult and old dogs exhibited anti-SARS-CoV-2 antibodies). No differences were observed for the sex and size of the animal. The only variable showing a greater exposure to the virus was the origin, as animals belonging to kennels had a higher prevalence (8.5%) than hunting dogs (0%) (Table 1). All feces from the sampled animals were tested with a one-step RT-real-time PCR protocol for the amplification of two viral genes. No sample tested positive for the two genes investigated.

**Table 1:** SARS-CoV-2 seroprevalence in the dog population of the Campania region: univariate analysis (chi-square) of potential risk factors (province, sex, age, bred, attitude, and size)

Factor	*n*	Positive	%	χ^2^	*p*
Total	112	5	4.5		
Province					
Avellino	19	0	0		
Benevento	11	1	9.1		
Salerno	36	1	2.8	3.6	0.46
Caserta	35	3	8.6		
Napoli	11	0	0		
Sex					
Male	67	2	3		
				0.85	0.35
Female	45	3	6.7		
Age					
Young	30	0	0		
Adult	55	2	3.6	4.28	0.12
Old	27	3	11.1		
Bred					
Mix	46	4	8.7		
				3.28	0.07
Specific bred	66	1	1.5		
Attitude					
Stray	59	5	8.5		
				4.7	**0.03**
Hunting	53	0	0		
Size					
Small	42	3	7.1		
Medium	58	1	1.7	2.1	0.34
Giant	12	1	8.3		

## Discussion

Since the first months after the outbreak of the SARS-CoV-2 pandemic, scientific and public health authorities have questioned the role of pets in the transmission of the virus (Teixeira et al. 2023). After a minor role in human transmission was established, various studies have assessed serological and molecularly positive domestic animals (Decaro et al. 2021a). The prevalences observed in the present study are very similar to those of other studies conducted in other geographical areas. For example, a seroprevalence of 4.94% and 6.46% were described in Korea and Hong Kong, respectively (Go et al. 2023). In Minnesota (United States), antibodies against SARS-CoV-2 were detected in a very low percentage of pet dogs (< 1%) (Dileepan et al. 2021). The same percentage was observed in Poland (only 3 out of 343 dogs had specific antibodies) and China (0.015%) (Wang et al. 2022; Pomorska-Mól et al. 2021). Only 0.41% of animals were positive during the early and mid-pandemic periods in Japan (2020) (Yamayoshi et al. 2023). In Italy, antibodies against N-protein were detected in 1.3% of pet dog serum in the same study area (Cardillo et al. 2022). In Spain, 3.59% of animals showed specific antibodies (Barroso-Arévalo et al. 2023). However, larger exposures have been described in Malaysia (24.5%) and in Lebanon (19.44%) (Khalife and Abdallah 2023; Tan et al. 2023). The differences observed between the various studies varied based on the test used (and the diagnostic performance in pets), epidemiological situation (sampling coincided or not with outbreaks, waves, and peaks), vaccination coverage in humans in the study area, type of virus (predominant variant in that specific period), and type of sampling (type of the selected dogs) (Guo et al. 2023). For example, when a second serology assay for virus neutralization (VNT) was used to confirm each positive sample, the seroprevalence was lower (Panei et al. 2022). This aspect has been described in several studies, including one carried out in Korea and Hong Kong, where prevalences ranged from 4.94 to 6.46% in ELISA and only 0.29% in VNT. A seroprevalence of 4.3% was described in Brazil, but only 0.4% when VNT was used (Jarrah et al. 2023). The same has been described in France when a western blot was used (Laidoudi et al. 2021).

True seroprevalence in pets could also be affected by COVID incidence in humans (de Souza Barbosa et al. 2022). Data from the integrated COVID-19 monitoring of Italian national public health organizations indicated 64 cases per 100,000 individuals per week, with a transmissibility index (Rt) of 0.9 (0.85–0.95). The variant of interest, EG.5, had a proportion of 39.4% (Aggiornamento nazionale relativo al periodo 18/09/2023—24/09/2023 dei dati della Sorveglianza Integrata COVID-19). For example, several studies found dogs from COVID-19-positive individuals were substantially more likely to test positive than those from COVID-19-negative households (Fritz et al. 2021). This aspect was evident in several studies that divided the population into “random” and “suspected” groups, and in some cases, it was also associated with the detection of the nucleic acids of the virus in the swabs of pets. The seroprevalence in those studies was between 30 and 40% (Mexico and the U.S.) (Fritz et al. 2021; Espinosa-Gómez et al. 2023).

The stool samples all tested negative for molecular investigations. Although it is more frequent to find molecular positivity in the oropharyngeal swabs of pets, some studies have also detected SARS-CoV-2 RNA in stool samples (Padilla-Blanco et al. 2022). However, no studies have been described regarding the diagnostic sensitivity and specificity of common real-time PCR protocols in dogs. This aspect could also depend on the health status of the sampled animals (in this case, apparently health). In fact, some studies have reported dogs with clinical signs (mild digestive and respiratory clinical signs) in Spain (Padilla-Blanco et al. 2022). From the dog involved in the case report described above, the variant was completely sequenced, and it emerged that it was SARS-CoV-2 Delta (B.1.617.2). Another almost complete sequencing from dog samples (nasopharyngeal and oral swabs) was reported in Botswana, and the variant involved was also Delta B.1.617.2, subsequently also described in Spain (Fernández-Bastit et al. 2021; Choga et al. 2023). In Spain, variant B.1.1.7 was sequenced from dog’s samples (Miró et al. 2021). In a study performed in Italy, variant B.1.177 was sequenced from oral and nasal swabs belonging to dogs (Anderson et al. 2020). The same variant has been isolated in a Spanish pet dog that presented hemorrhagic diarrhea (Padilla-Blanco et al. 2022). For this reason, it is possible to suspect that there is also a predisposition in animals to be infected by some variants and, therefore, to show symptoms and eliminate the virus with their own fluids. A meta-analysis study found that both molecular and serological prevalence in the general pet population were typically less than 5% but increased to more than 10% when COVID-19 positive persons were present in the household (Meisner et al. 2022; Guo et al. 2023). In a large-scale study carried out in Chile, in which approximately 700 dogs were tested by real-time PCR, approximately 6% were positive (Agüero et al. 2024).

Although there are numerous studies describing the exposure of dogs to SARS-CoV-2, studies that have evaluated the risk factors associated with greater exposure are few. The present study is among these, and, although with limited sampling which reduces the chance of detecting a true effect, it highlighted that shelter dogs had a higher seroprevalence. A study performed in Brazil focused on the difference in SARS-CoV-2 exposure among sheltered, foster home, and owner dogs and confirmed the results of this study, describing a seroprevalence of 20.22% in shelter dogs (Nilsson et al. 2024). Moreover, in the same study, age was also recognized as a risk factor, with adult dogs having a 4.16 chance of positivity (unlike the present study). Other significant factors (not assessed in the present study) were high population density among dogs and humans and repeated COVID-19 exposure through human–dog interactions (Nilsson et al. 2024). On the other hand, a recent study performed in northern Italy did not find any correlation between kennel dogs and higher seroprevalence (Bellinati et al. 2023). However, it is still intriguing to consider the dynamics of infection transmission to kennel animals. Were the dogs infected before entering the shelter because of environmental contamination? Or, (a more plausible scenario), were the animals exposed to the virus as a result of interactions with infected operators? Given the promiscuity and living circumstances of kennel dogs, was the virus possible to spread to others? Although years have passed since the infectious potential of SARS-CoV-2 in dogs was proven, this concern is still unanswered (Murphy and Ly 2021).

SARS-CoV-2 has generated hundreds of outbreaks in various animal species across multiple nations. It is necessary to continue monitoring the spread of this virus in domestic and wild animals, notably those more predisposed and with closer contact with humans as dogs.

## Conclusions

Pets have been and continue to be predisposed to exposure to SARS-CoV-2 due to their relationship with humans. In this study, the seroprevalence of shelter dogs in a region of southern Italy was evaluated. The results obtained highlight the circulation of SARS-CoV-2 in this population of dogs, leading to the hypothesis that contact with infected operators is sufficient to transmit the infection to dogs. This is a small-scale study, and because of the limited sampling, it is unable to determine the significance of the several risk variables involved in the transmission of SARS-CoV-2 to dogs. In recent years, several aspects of the transmission of this virus to pets have been clarified, while others have yet to be clarified, such as the dynamics of intraspecific transmission. Future studies are needed to clarify these concepts and continue the surveillance of this reverse zoonosis in domestic animals.

## Data Availability

No datasets were generated or analysed during the current study.
